# Inter-specialty collaboration in the formalization of a new foregut subspecialty

**DOI:** 10.1371/journal.pone.0262019

**Published:** 2021-12-30

**Authors:** Hannah Vassaur, Peter Martelli

**Affiliations:** Sawyer Business School, Suffolk University, Boston, Massachusetts, United States of America; Sapienza University of Rome: Universita degli Studi di Roma La Sapienza, ITALY

## Abstract

Historical context and converging market conditions present a unique opportunity to study the factors contributing to the formalization of foregut, or upper gastrointestinal, surgery as a new subspecialty in United States healthcare. The aim of this study was to examine the justifications of those pursuing the formalization of a foregut subspecialty and to extract lessons for healthcare leaders on the evolving relationships between competing providers. This was a qualitative, cross-sectional study consisting of interviews, secondary survey data, and observation of society meetings between October 2018 and June 2019. Thirty interviews with healthcare professionals were conducted, transcribed, and analyzed for themes using qualitative coding software. Themes were correlated with observational field notes and archival data and compared against established social theories on professions and medical specialization. Analysis revealed that traditionally competing surgeons and gastroenterologists articulate a professional need to qualify foregut surgical expertise based on superior knowledge and outcomes, to define the allocation of certain tasks and procedures, and to foster collaboration across specialties. Converging market conditions precipitate individual and organizational decisions to pursue formal specialization. Participants in the formalization of this subspecialty should consider the history of professional competition and turf wars to achieve meaningful collaboration. Advocacy for multi-specialty societies and organizational movements could be a model for reduced conflict in other specialties as well.

## Introduction

Diseases of the foregut represent a large and growing burden in the United States (US) and a significant source of controversy among treating providers. The term “foregut” is typically used by clinicians to reference the beginning portions of the gastrointestinal tract, including the esophagus, stomach, and first portion of the small intestines. For example, one such disease, gastroesophageal reflux disease (GERD), affects an estimated 10–20% of the adult population in the US [1]. Persistent foregut complaints and disease progression result in millions of visits annually to primary care providers, gastroenterologists, surgeons, and emergency rooms in the US [2], representing significant cost to the system [3]. Such disease trends have spurred significant innovation in this disease space over the past decade by both venture capitalists and large medical device companies [4, 5].

Consequently, providers can select from a variety of changing tools in their management of foregut diseases and must acquire new skills to keep pace with the market. New technologies include advanced diagnostic therapies that detect early disease progression, videoendoscopic therapies that mitigate surgical intervention, and minimally invasive surgical therapies to alleviate advanced symptoms or cure malignant disease [4]. Both medical and surgical societies publish patient management guidelines at regular intervals [6], while expert panels and working groups release their own consensus statements on clinical algorithms in various journals [7]; not all are in concordance. In the spring of 2018, the Society of American Gastrointestinal and Endoscopic Surgeons (SAGES) established a new Foregut Task Force to “determine standards to optimize patient safety” in the management and treatment of foregut diseases [8]. In 2019, the same society published *The SAGES Manual of Foregut Surgery*, with an online introduction describing its comprehensive review and expert commentary aimed “to clarify controversies in the field” [9].

Concurrently, a new multi-disciplinary society, the American Foregut Society (AFS), emerged, recruiting membership throughout 2018 and hosting its inaugural meeting in March 2019 [10]. The advocacy for this new, multidisciplinary specialty society coincides with other noteworthy provider trends, such as the advent of a new “foregut” fellowship designation and the implementation of esophageal and foregut disease service lines, or “Centers of Excellence” in hospitals [11]. These evolving provider and organizational trends have afforded a unique opportunity to examine the development of the new foregut surgical specialty parallel to its emergence. Understanding the context, provider attitudes, as well as historical social patterns may allow healthcare administrators to influence subspecialty progress toward provider collaboration rather than competition.

Professional competition over medical turf, procedures, and specialties has been described in the sociology literature for decades, with each new theory reflecting the evolution of healthcare during that period [12]. While many of the early theories focus on the client-professional relationship and common characteristics that define a professional, theories have since shifted toward understanding the motivations of professionals that might result in conflict [13]. Eliot Freidson, a key influencer in this movement, describes the “privileged position” of professionals, including physicians, which contributes to competition over the rewards associated with such status [14]. Freidson contends that physicians traditionally exert collegial control via collective, self-regulation because of the level of autonomy expected of professional decision-making, and in so doing, they ensure their continued professional dominance [15]. Critics of Freidson’s theories point out that physicians have become increasingly beholden to bureaucratic processes, such as credentialing or employment by hospitals, because of the expansion of capitalism in the medical profession [16]. When examining the profiles of professionals in the hospital, organizational theorists describe the profile of physicians as competitive, occasionally to the point of sabotage, which can be reinforced by training systems that emphasize individual performance and by the social reward of prestige [17].

Competition over disease space ownership has historically resulted in the emergence of new, regulated specialty identities, as in the case of obstetrics [18], or persistent conflict between competing specialists, as with gastroenterologists and surgeons [19]. The dynamic relationship between the latter was particularly exacerbated by gastroenterologists’ development and champion of the videoendoscope, which disrupted the traditional workflow and dominant position previously held by surgeons [20]. Sudden innovation can trigger intra-specialty and inter-specialty turf warring by causing a professions system disturbance [13], which then leads to formal attempts to establish control over certain tasks [21]. In such times of heightened conflict, emerging specialists use claims of superiority in knowledge, experience, or outcomes to legitimize their movement toward a position of dominance [20, 22].

Structural position in the workflow is a critical factor in determining the outcomes of jurisdictional disputes, or attempts to control certain tasks, procedures, or anatomy [23, 24]. Referral-dependent professionals, like surgeons, may have trouble exerting regulative or normative control over their profession [13]; in such cases, timing can be the deciding factor for turf war outcomes [20].

Based on our understanding of these theories, we hypothesized that the quickly evolving advancements in the foregut disease space are driving a faction of surgeons to seek regulative control over the foregut subspecialty. Our primary research question was as follows: What individual provider attitudes and organizational behaviors are contributing to the formalization of a foregut sub-specialty?

## Method

### Study design

This was a qualitative, cross-sectional study consisting of interviews, archival and survey data, and non-participant observation, intending to explore the provider attitudes, behaviors, and market conditions that are contributing to the creation of a new foregut subspecialty.

The primary author, a clinician with experience in the subject matter, led all interviews. Her background includes work as a certified physician assistant in general and bariatric surgery, a Master of Business Administration with healthcare emphasis, and consultant for reflux and endosurgery device manufacturers. Due to her role in the medical device industry working with hospital administrators, the primary author had a preexisting collegial relationship with many of the interviewees; they were formally notified of the purpose of the research as related to graduate level studies in healthcare management and pursuit of publication of these findings, independent of her professional employment. The second author is an Associate Professor of Healthcare Administration at Suffolk University with a PhD in Health Services and Policy Analysis from UC-Berkeley. He served as the first author’s independent study preceptor, overseeing development of the interview guide, participating in limited data collection, reviewing all transcripts, and supporting data analysis and manuscript development. Participants were healthcare professionals purposively selected based on their self-identification as esophageal or foregut specialists, known participation in an esophageal or foregut focused practice, or direct professional involvement with such providers in a healthcare setting. They were approached face-to-face at a medical conference or via email script by the lead author. Interview data was coupled with field notes from observation of the inaugural AFS conference, archival review of working documents, and descriptive analysis of data from the first AFS member survey, made available to the researchers as a secondary data source by AFS (Table 1).

**Table 1:** Survey results from AFS inaugural meeting.

**Multiple Choice Questions**	**n**	**Answer Choice**	**Responses (Percentage)**
1. I am a:	216	General Surgeon	139 (64.4%)
		Thoracic Surgeon	18 (7.9%)
		Gastroenterologist	43 (19.9%)
		Allied Health Practitioner	7 (3.2%)
		Other	17 (8.3%)
2. What type of practice setting do you work in?	214	Academic practice, predominant clinical	74 (34.6%)
		Private practice plus clinical research	63 (29.4%)
		Private practice without research	51 (23.8%)
		Academic practice, predominant research	9 (4.2%)
		Other	19 (8.9%)
3. What portion of your practice involves Foregut Disease?	212	0–25%	17 (8.0%)
		25–50%	58 (27.4%)
		50–75%	62 (29.2%)
		75–100%	75 (35.4%)
4. Have you done specialty training (fellowship) in foregut disease?	212	Yes	123 (58.0%)
		No	89 (42.0%)
5. Number of years in practice	212	In training	3 (1.4%)
		0–5	35 (16.5%)
		6–10	35 (16.5%)
		11–15	34 (16.1%)
		More than 15	105 (49.5%)
6. Describe the main/primary institution where you work:	211	No residents or fellows	73 (34.6%)
		Fellowship trainees	58 (27.5%)
		Resident only, no fellows	46 (21.8%)
		Specific foregut fellowship training as >50% of total training	32 (15.2%)
		No Fellows but interested in developing a program	19 (9.0%)
7. What is your level of interest in AFS?	206	I will participate in any way to make it flourish	74 (35.9%)
		Very interested in participating including committees	91 (44.2%)
		Will definitely come to meetings annually but not able to beyond that	27 (13.1%)
		Maybe come to an annual meeting—just want to support a good cause	14 (6.8%)
8. Organizations (such as the AFS) should get involved in making training/credentialing where there is evidence to support specific criteria (i.e. RFA/ Anti-reflux procedures).	200	Current training programs and credentialing recommendations are adequate	29 (14.5%)
		Individual institutions should police the privileging/credentialing for these procedures.	47 (23.5%)
		There is sufficient evidence to support common procedures (RFA, Lap Antireflux procedures) having specific training / credentialing criteria (such as case volume).	124 (62.0%)
9. What percent of a provider’s clinical practice should be foregut for someone to really be a specialist in this arena:	200	<25%	14 (7.0%)
		25–50%	82 (41.0%)
		Over 50%	93 (46.5%)
		Over 80%	11 (5.5%)
**Ratings Questions**	n	Answer Choice	Weighted Average (0–5 Scale)
10. With the ultimate goal of improving patient care, answer each goal from 0 (Not important from my standpoint) to 5 (Critical) in terms of what is important FOR YOU that AFS achieve:	206	A forum to share thoughts among like-minded people	4.5
		Meetings focusing at a high level just on foregut disease	4.4
		Bring greater collaboration between disciplines	4.3
		Establish guidelines to foster specialization	4.0
		Establish foregut as a specialty	3.6
11. Rate your interest level in each topic listed below (0-BORING to 5-FASCINATING)	206	Management of GERD, surgical and medical	4.7
		Motility Disorders of the esophagus and stomach	4.3
		Barrett’s, Dysplastic Barrett’s, Early esophageal neoplasia	4.2
		Foregut neoplasia	3.5
		Inflammatory disorders of the esophagus (eg EoE, lymphocytic esophagitis)	3.0
		Endoscopic & surgical bariatrics	2.7

**Fig 1:**
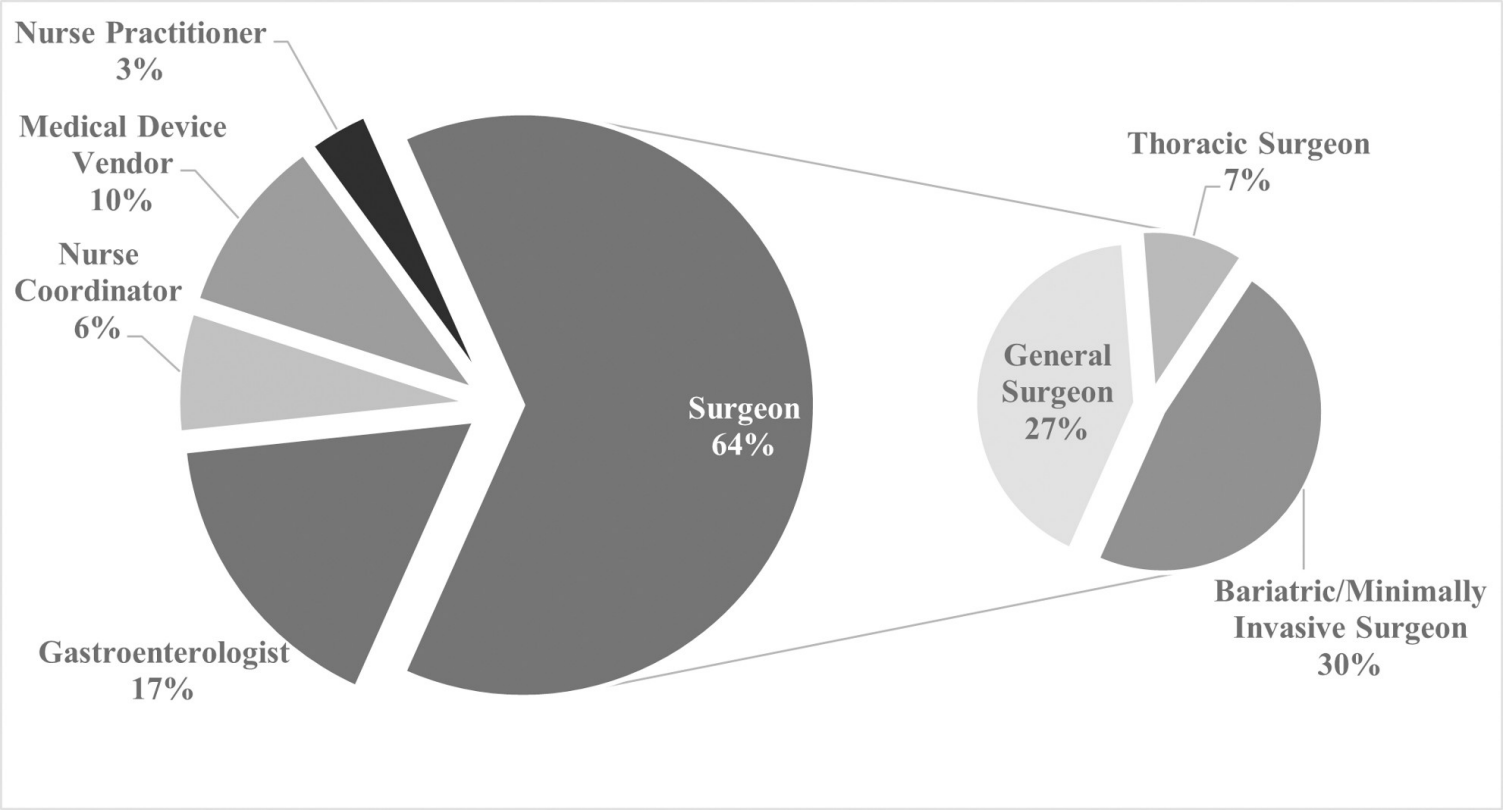
Distribution of interviewees by role.

Semi-structured interviews were conducted with 30 professionals from October 2018 to June 2019, ceasing at theoretical saturation (Fig 1 and Table 2). Interviews followed an interview guide (S1 Appendix) and lasted 15–35 minutes each, with no repeat interviews required and only researchers and participants present. Interviews were audio-recorded and transcribed verbatim; they were not returned for comment or correction to participants. Research procedures were approved by the Institutional Review Board at Suffolk University (Protocol #1295907–2), and informed written consent was obtained from all participants. No interviewees withdrew after providing written consent to participate, and participant information was anonymized.

**Table 2:** Additional interview characteristics.

**Interview Setting (n = 30)**	
**American College of Surgeons Conference**	2 (7%)
**SAGES Committee Meetings**	4 (13%)
**AFS Inaugural Meeting**	6 (20%)
**Other Conference**	2 (7%)
**Physician’s Office**	1 (3%)
**Phone**	14 (47%)
**Email Correspondence**	1 (3%)
**Provider Practice Setting (n = 27)**	
**Academic Hospital Employed**	12 (44%)
**Community Hospital Employed**	9 (33%)
**Community Hospital–Private Group**	6 (22%)

### Data analysis

Content analysis of the qualitative interview data was performed using N*Vivo v12. With the help of two research assistants, the authors generated broad codes based on the social theory of professions and independently assigned them to a subset of five transcripts. This subset was then closely reviewed to reconcile coding assignments, generate focused codes, and develop supplementary codes from emergent concepts (Table 3). Coding of the full set of transcripts was performed by the lead author, and a second subset was fully coded by both authors to reduce bias, demonstrating high interrater reliability (Cohen’s kappa = 0.8487). Themes in the coded transcripts were developed and compared with observational field notes and archival data; participants were not approached for feedback on the findings.

**Table 3:** Coding guide.

Code	Subcode	Description	Example terms
**Claims of Superiority**	Cognitive abstraction	Inter-professional competition strategy based on knowledge or superior training	“my training” “my knowledge” “special interest in this subject” “expert”
	Age/Tenure	The conflict (i.e. opposition to or support of specialization) is intergenerational and cohort based.	“my tenure” “my experience”
	Effective results	Inter-professional competition strategy based on superior outcomes/results	“my outcomes” “my patients do better” “more volume equals better outcomes”
	Collective Altruism	Professionals are expected to provide their specialized knowledge/training to the clients in order to maintain their status and legal protections.	Patient-centric language “Service to community” “for the benefit of patients/the disease space”
**Professional Dominance (status)**	Status	The professionals are competing for status and the rewards associated with status, i.e. “privileged position”.	“specialists” or other special labels “accreditation” or “setting standards” in order to be labeled
	Delegitimation threat	Complications can destroy the reputation of a new technology and its users, threatening their control.	“Protect reputation”
**Task Structure**	Structural dependence of surgeons	Surgeons have a downstream position in the specialty workflow that makes them *dependent* on referrals from GIs, PCPs, and others.	“referrals”
	Symbiotic interdependencies of the specialties	Surgeons and GIs have a mutually beneficial relationship; both specialties are incentivized to maintain friendly relationships.	“mutual benefit” “sharing patients” “trust”
	Bridging strategies	Professionals negotiate *between* organizations to change control over their environment and add legitimacy to their movement.	“work with” “work together” With GIs or with other societies, like SAGES
**Professional Jurisdiction (control)**	Inter-specialty anatomical turf warring	Competition between specialties, i.e. GIs and Surgeons to control specific anatomy/tasks/procedures	Conflict between surgeons and GIs “Bias” “disagree”
	Intra-specialty anatomical turf warring	Competition within a specialty, i.e. foregut surgeons and general surgeons to control specific anatomy/tasks/procedures	Conflict between general surgeons (or societies) and foregut surgeons (or AFS) “Foregut” “General surgeons”
	Monopoly closure	The professionals are attempting to establish a monopoly over specific procedures.	“volume” “practice focus” “majority”
	Task jurisdiction or “core skills”	The clear definition of a provider’s “core skills” helps to communicate to others what their jurisdiction is, and how that is important in the physician workflow. Proceduralists like surgeons/GIs may stake claim over specific procedures, technologies, or anatomy.	Specific procedures or specific anatomy mentioned
**Professional Interest**	n/a	The professionals are driven by subject matter interest and need for professional fulfillment.	“interest” “fun” “satisfying”
**Market Timing**	Economic drivers	Gaining payment for new procedures, building a practice, or increasing revenue in the hospital may be accelerating the movement toward specialization.	“insurance” “revenue”
	Local market conditions	Each physician’s local market need/opportunity may influence desire to specialize.	“my practice” “my market”
	Emerging technology	Technological advancements in recent years may be promoting specialization by creating new tasks for specialists to claim and increasing the perceived need for training.	“new technologies” “recent advancements”

## Results

The surgeon interviewees articulate a professional need to qualify foregut expertise based on superior knowledge and outcomes, to foster collaboration across specialties, and to define the allocation of certain tasks and procedures in this field. Non-surgeon participants confirm these themes in their descriptions of surgeon colleagues and evolution of the space. Current specific market conditions are discussed as precipitating individual and organizational decisions to pursue formal specialization.

### Claims of superiority: Qualifying expertise

The respondents suggest that only a small number of general, thoracic, and bariatric surgeons receive specific training in the diagnosis and treatment of esophageal disease and have the surgical volumes to back their claim of superior experience.

Every procedure is technically demanding. It requires precision.–R7

It’s not something I just do once a month, so I think volumes speak volumes in this case.–R9

Similarly, one faction of gastroenterologists is noted to have particular skill in physiologic testing and interventional therapy for esophageal disease.

It requires dedication to learn how to interpret the testing. Right? It’s not something you can read about and then the following day you’re good to go.–R6

They have additional training in the specialty trainings, diagnostics… they’re committed to the disease in a way that, that propels us forward, I think.–R19

Many respondents clarify that completion of a minimally invasive surgery (MIS) fellowship does not necessarily equate to foregut expertise. In the AFS membership survey, 52% of respondents believed that greater than half of practice volume should be devoted to foregut surgery to “really be a specialist” (Table 1). Conversely, the SAGES proposed criteria for Advanced GI/MIS Fellowship requires only 20 foregut cases during fellowship [25], yet a published survey of MIS trainees reveals that 52% of MIS fellows will identify themselves as “foregut surgeons” upon graduation [26].

Second to specialized knowledge, surgeons and gastroenterologists emphasize better outcomes as evidence of superiority, suggesting a collective altruistic concern for patient welfare and drawing on the literature to back claims.

I believe in this day of super-specialization, you can’t dabble. If you dabble and do less than a certain number of cases a year, your outcomes are not going to be as good.–R1

There is increasing evidence on the surgical side that foregut disease care is better if it’s specialized. And that not every Tom, Dick, or Harry ought to be doing this.–R2

### Professional dominance: Protecting reputation

In conjunction with superior outcome claims arise themes of reputation and restriction of professional labels.

The outcomes have been poor, the complication rates have been high, and it has gotten foregut a very bad name. Both in the medical community as well as, more importantly, the patient community.–R4

Not surprisingly, many surgeons suggest the role of a new society to restrict the labeling of “Specialists” and “Centers of Excellence” to those who meet certain criteria.

We’ll have to get to a point where if you want to be part of a national system of being recognized as being excellent, then you’re going to have to prove it and you’ll have to set up criteria for people to do so.–R10

In fact, the secondary goal listed in the mission statement of the American Foregut Society is “to foster research that will culminate in the development of benchmarks for excellence” [27]. The majority of membership confirmed the necessity of credentialing criteria in the AFS survey (Table 1).

### Task structure: Altering workflow and dependency

The dependence of the surgeons, both on referrals and expensive healthcare resources, is discussed in 60% of interviews, with one surgeon jokingly dubbing their position as “the end of the food chain” (R18). Their position within the workflow seems particularly dependent on the gastroenterologists, which at times affects which procedures surgeons are willing to offer.

I’m competing against the people who are going to send me the reflux patients. So basically, I don’t scope them because they’ll scope them. Alright so in other words, don’t bite the hand that feeds you.–R8

This same respondent also suggests that despite his dependence, surgeons and gastroenterologists can structure a symbiotic collegial relationship benefiting both parties. This sentiment of a mutually beneficial working relationship is noted by 57% of respondents, who describe sharing patients, complementary services, “common goals”, and “collaboration” in developing their local program. Collaboration and its synonyms are used in over one third of the interviews.

Collaboration is particularly noteworthy in connection with AFS, whose mission statement begins with the aim “to help guide both the diagnosis and management of Foregut disease through collaboration between Gastroenterologists and Foregut Surgeons” [27]. The term was also used frequently at the inaugural AFS meeting by both gastroenterologist and surgeon speakers, along with the terms “align”, “consensus”, and “team”. Intentions to improve collegiality are noted frequently throughout the interviews.

There’s a real attempt to… create a collegial environment between gastroenterology and general or foregut surgeons.–R12

While some respondents suggest leveraging the influence of other surgical societies in accomplishing goals, most draw distinction between the AFS and existing societies in its aim to influence the collaboration between medical and surgical foregut specialists.

There isn’t any way that SAGES can bring the same type of focus to those particular problems that I just mentioned, as this society can.–R16

### Task jurisdiction: Defining turf

Despite the advocacy for collaboration, however, remnants of the historical inter-specialty turf war between surgeons and gastroenterologists persist, with more senior surgeons referencing it specifically.

I believe that general surgery and gastroenterology have been slow to get out of silos… and there’s too much turf. Endoscopy is a great example.–R8

This historical turf war over endoscopy, ongoing disagreements in disease management, or professional bias are referenced in 70% of interviews, and the resulting conflicting guidelines between surgical and gastrointestinal societies is mentioned as an area to be addressed by a multispecialty society.

They evaluate the diseases of the foregut, and address its treatment from a medical perspective, whereas we have a tendency to address it from a surgical perspective. Those are the relative tools that we have.–R16

Evidence of intra-specialty turf warring appears in the interviews as well, with references to the delineation of general surgeons versus foregut surgeons.

Just because you’re a surgeon, just because you’re a gastroenterologist doesn’t mean you’re qualified to be a foregut surgeon and be a specialist in that field.–R12

Surgical turf warring is highlighted in discussions of societies, particularly when respondents are prompted to describe the role of SAGES compared to the new AFS.

But SAGES still wants to consider foregut to be part of general surgery…they don’t promote it as a specialty. They can’t…because they represent general surgery.–R2

Ultimately, the conflict between specialties, within specialties, and among societies regards qualified ownership of tests or procedures in the continuum of care.

Surgeons need to do this. But if they can’t get the experience, and that means accreditations, that means credentialing, that means then proving that they can, you know, safely perform these things in their hospitals throughout the United States.–R10

### Professional interests and collegiality

In qualifying their colleagues, the interviewed surgeons were quick to distinguish those who have the “interest” to specialize in esophageal or foregut care. In fact, “interest” and its synonyms are used in 63% of the interviews.

I’ve always had an interest in anti-reflux operations and my practice sort of was headed that way in any case and so I decided to just stop performing general surgery and focusing on foregut.–R7

Particular “interest” is often discussed in conjunction with commitment to the disease space in some form, whether in procedural volume, research, or training; “dedication” is mentioned in 9/30 (30%) of interviews, often as a requirement for labeling oneself a foregut or esophageal specialist.

A dedicated interest in expertise in adopting new technologies, pioneering new treatments in this area would be the main thing I would look for.–R26

Common interest appears to be a prompt for collegiality and partnerships in the creation of AFS and new Centers of Excellence. Results from the AFS survey indicate that membership highly rank “a forum to share thoughts among like-minded people” as “Critical” in the goals of AFS (Table 1). Common interest and a desire for collegiality is also evidenced in the success of the SAGES online social media forum dedicated to foregut surgery [28].

### Market timing

Some economic drivers in the market, such as seeking insurance approval for new procedures, generating hospital revenue, and acquiring a volume monopoly over certain procedures, present as themes in the interviews. Financial concerns, however, are not discussed as elaborately as qualifying expertise when examining the needs of space; instead, these themes often arise only when specifically prompted by the interviewer.

INT: Is, is the hospital invested in having those tests done? I mean, is it lucrative?

RES: I think so. Uh, I don’t know the ins and outs of the cost and you know the, uh, reimbursement.–R4

Local market conditions appear to influence individual decisions to specialize or to open a center with special foregut dedication, often in combination with professional interest.

And my focus has certainly over the last two years evolved from general surgery into primarily foregut specialization…but with the departure of [another surgeon], there was a huge patient volume that was in the midst of a workup and also needed follow up. So I inherited all of those patients.–R15

Recent advancements in diagnostic and therapeutic technologies are identified as the major catalysts for maturation of the space and drivers for specialization. “Technology” is referenced by 36% of interviewees. Four new therapeutic procedures are mentioned by name 67 times in the interviews; robotic surgery is also mentioned by five interviewed surgeons.

You need specialization because there are more tools. Both in the surgical arena with different anti-reflux procedures.–R2

The described market conditions, as well as references to historical dynamics between surgeons and gastroenterologists, suggest a critical juncture in cross-disciplinary relations as these providers pursue the formation of this subspecialty.

## Discussion

The results of this study indicate that converging market conditions have created volatility among professions, leading to formal jurisdictional claims by a cohort of physicians. Based on the themes analyzed, the surgeon specialists aim to protect the reputation of and to exert professional dominance over their evolving field by collaborating with a select group of their traditional competitors, with whom they identify as having common interests.

Precipitous innovation in this disease space seems to motivate the self-described specialists to seek exclusive control over certain procedures, thereby enhancing their claims to superiority in this arena. This method of professional differentiation based on unique knowledge is a concept described by Andrew Abbott as “cognitive abstraction” in his discussion of professional competition [22]. The secondary emphasis on superior outcomes suggests a concern over the dilution of reputation that could occur with poor patient outcomes, harkening to Zetka’s description of the “delegitimation threat” in the early days of the surgical laparoscope [20]. Promotion of improved outcomes also implies a collective concern for the patient’s well-being, perhaps an effort to fulfill their tacit professional contract with society while protecting professional reputation [29].

Using superiority claims, these professionals seek formal, regulative distinction between foregut specialists and others. Intra-disciplinary competition, evidenced by use of terms like “dabblers” and references to inadequate training, seems a significant impetus for the creation of a new specialty society. The results from the AFS survey indicate that most members want a professional society to outline foregut credentials, perhaps a preemptive move to self-regulate and maintain collective autonomy (Table 1) [14]. Legal definition or differentiation of foregut expertise will be difficult to establish on the collective rhetoric of superior knowledge alone [20]. Well-established surgical societies may resist formalization of foregut credentials under the abstract claim of superior knowledge, as evidenced in the society tensions alluded to by respondents. Zetka’s theories suggest that soliciting support of other organizations, other professionals, or the patients themselves will be more efficient than claims of superiority in establishing turf dominance. Thus, “bridging” their movement with organizations like SAGES and the American College of Surgeons may be required to legitimize the efforts of the new society and its constituents [30].

The self-described foregut surgeons are, however, deploying bridging strategies with gastroenterologists in effort to exert some control over their environment [31]. While it is possible that some surgeons have learned to espouse the importance of collaboration to curry favor with colleagues, the behaviors manifested by these interviewees, such as partnership in the creation of Centers of Excellence and inclusion of gastroenterologists in a new society development, suggest a legitimate interest in promoting team-based care within their profession, albeit with some benefit to their dependent position.

Unlike the jurisdictional contests typically discussed in the literature [18, 20], foregut surgeons stake claim over specific surgical tasks while relinquishing jurisdiction over others in order to maintain positive relationships with a key referral source and traditional competitor. Given the surgeons’ structural dependence in the referral pathway, downstream from gastroenterologists, purposeful collaboration with gastroenterologists may prove more successful than claims to cognitive superiority in establishing dominance over the surgical component of the subspecialty, effectively cutting off referrals for general surgeons. Interestingly, while their structural dependence is readily apparent and admitted, foregut surgeons also claim collegiality and a symbiotically beneficial relationship with GIs. Propagating this rhetoric may enhance the more formal professional bridging strategies of including gastroenterologists in their newly forming society and in multi-specialty Centers of Excellence.

The interviewed surgeons are quick to distinguish their gastroenterological colleagues who have foregut interest from those who do not, elevating them to a similar professional status within the boundaries of the foregut subspecialty. In the absence of a formal “foregut” specialty in prior years, and few programs devoted to specific training, many interviewed surgeons and gastroenterologists have chosen this area of focus through evolving, real-world preference; a desire to protect its reputation may foster comradery amongst traditionally competing medical and surgical providers, rather than overt antagonism. The creation of multi-specialty Centers of Excellence exists as a concrete, team-based goal relying on group rather than individual performance to realize its full value in the market.

The study results demonstrate some incongruity between a desire for collaboration and the bias against non-surgical colleagues. Of note, surgeon representation at the inaugural AFS meeting was nearly three times greater than that of gastroenterologists. A recent article published in a gastrointestinal journal, co-authored by a gastroenterologist and a surgeon, discusses that though technological advancements in both specialties have begun to “erode the traditional turf” between these two parties, differences persist in protocols, which feed an underlying competitive spirit [32]. Shared bias, ingrained by siloed physician training structures and decades of competition [17, 33], may result in skepticism from gastroenterologists and threaten further recruitment to the cause.

Consistent with theories discussed above, precipitous innovation in this disease space seems to motivate the self-described specialists to seek exclusive control over new procedures, thereby enhancing their prior claims to superior knowledge. Prior alimentary turf losses to gastroenterologists with the advent of the endoscope, the historical reputation of foregut surgery, and recent, rapid innovation converge to create a critical moment in the foregut subspecialty, in which surgeons and gastroenterologists can influence the outcome of this movement by choosing to collaborate and overcome professional differences.

Strengths of the study include the duration and quality of interviews obtained, which resulted in detailed transcripts for interpretation. Limitations of the study included its cross-sectional design, which may have resulted in bias particular to the types of providers most willing to discuss evolution in the space. It was more difficult to elicit interviews from providers that have not chosen to join the emerging society to gain their perspective on the changes, though a few are represented in the data. The study is further limited by its US focus; there is currently an international discussion around specialization, as evidenced in the literature and by the recent inaugural meeting of the European Foregut Society Meeting in Vienna, Austria [34]. A larger study collecting international data would reveal the potential global impact of these observations.

## Conclusion

Self-identifying foregut surgeons collaborate across traditional competing specialties to establish professional dominance as sub-specialists in an increasingly focused US healthcare system. The manifested cross-disciplinary dialogue could advance quality improvement mechanisms and guideline alignment in this particular disease space nationally and internationally. Healthcare leaders pursuing the development of formal collaborative movements between these specialists, such as Esophageal Centers of Excellence or Heartburn Programs, should consider the social theory behind professional competition and historical outcomes of turf wars if they hope to achieve successful collaboration instead of further division between competing physicians.

## Supporting information

S1 Appendix Interview guide. (DOCX)

S2 Appendix COREQ checklist. (PDF)
