# Supplementary material for: Inter-specialty collaboration in the formalization of a new foregut subspecialty
Source: PLoS One. 2021 Dec 30;16(12):e0262019. doi: 10.1371/journal.pone.0262019 (PMC8718094; doi:10.1371/journal.pone.0262019)
Supplement: S1 Appendix — (DOCX) [file pone.0262019.s001.docx]

**Introductions**

Hello, and thank you for agreeing to talk with me today. Your responses will be kept confidential and no reference will be made to your name or any other identifying information. Your identity will be disguised in any publications or presentations. **Is this okay with you?**

Your participation is voluntary, and you may stop the interview at any point or refuse to respond to any question you don’t feel comfortable answering. We’re would like to speak with you for about 30 minutes. **Does this still work with your schedule?**

We want to ensure that we accurately capture your responses to these questions. If you agree, we would like to audio record and transcribe this interview for our later analysis. If you choose not to be audio recorded, we will take handwritten notes of the interview. **Is it okay to record this interview?**

I am going to ask you some questions about your impressions of. My goal is to understand your perspective in detail. **Do you have any questions before we begin?**

**Questions and probes**

1. Please tell us a little about this clinic or hospital.
   1. Probe on population characteristics, staff size, range of services offered, catchment area (size, urban/rural), how care is paid for
2. Operationally, how are patients with esophageal disease managed now
   1. Involved provider titles, diagnosis, treatment, follow-up
   2. Tell me about a time that your current system operated sub-optimally
   3. How could it be organized better?
   4. From the hospital’s perspective, what method could optimize revenue?
3. Does your hospital or practice currently have a service line or brand specifically devoted to the Foregut (esophagus + stomach, Heartburn, GERD, Reflux and/or swallowing)? (if no, proceed to question 4)
   1. Please tell us about its history and what roles were involved in its creation.
   2. What were the critical challenges/barriers in implementing this service line?
   3. Involvement of a Strategy Officer or outside Advisory Board?
   4. Proceed to question 6
4. Is your hospital planning to add such a service line or brand within the next 12 months? (If no, proceed to question 5)
   1. Please tell us about this process and what roles are involved in its creation.
   2. What do you consider to be the critical challenges/barriers in implementing this service line?
5. If not, why not?
6. What is your level of involvement with medical professional societies (e.g. SAGES, ACS, ATS, ACG)?
   1. In your opinion, how are these societies affecting the care of the Esophagus/Foregut specifically?
   2. What is your impression of the existing protocols (or clinical guidelines) for patients with esophageal/foregut disease?
7. What would you think of this disease having its own designation/specialty?
   1. If there were a new society, what would it do?
   2. What do you think gives someone expertise in this specific area?
   3. What could be the clinical and/or economic impact for creating a new specialty?
8. If you could change *one thing* tomorrow to make Esophageal/Foregut care better, what would you do?
9. Is there anything else you would like to share?
